# Genotoxicity assessment of titanium dioxide nanoparticle accumulation of 90 days in the liver of *gpt* delta transgenic mice

**DOI:** 10.1186/s41021-020-0146-3

**Published:** 2020-02-10

**Authors:** Tetsuya Suzuki, Nobuhiko Miura, Rieko Hojo, Yukie Yanagiba, Megumi Suda, Tatsuya Hasegawa, Muneyuki Miyagawa, Rui-Sheng Wang

**Affiliations:** 1 Division of Industrial Toxicology and Health Effects Research, National Institute of Occupational Safety and Health, 6-21-1 Nagao, Tama-ku, Kawasaki, Kanagawa 214-8585 Japan; 2 Present address: Graduate School of Biomedical and Health Sciences, Hiroshima University, Hiroshima, 734-8553 Japan; 3 Present Address: Department of Health Science, Yokohama University of Pharmacy, Yokohama, 245-0066 Japan; 4 Division of Human Environmental Science, Mount Fuji Research Institute, Yamanashi Prefectural Government, 5597-1 Kenmarubi, Kamiyoshida, Fujiyoshida, Yamanashi, 403-0005 Japan; 5 Present Address: Department of Sport and Medical Science, Faculty of Medical Technology, Teikyo University, Hachioji, Tokyo, 192-0835 Japan

**Keywords:** Titanium dioxide, Nanoparticles, *gpt* delta mice, Mutation frequency

## Abstract

**Backgound:**

A variety of in vivo and in vitro studies to assess the genotoxicity of titanium dioxide nanoparticles (TiO_2_ NPs) have been reported, but the results are inconsistent. Recently, we reported that TiO_2_ NPs exhibit no genotoxic effects in the liver and erythrocytes during a relatively brief period following intravenous injection into mice. However, there is no information about long-term genotoxicity due to TiO_2_ NP accumulation in tissues. In this study, we investigated the long-term mutagenic effects of TiO_2_ NPs and the localization of residual TiO_2_ NPs in mouse liver after multiple intravenous injections.

**Results:**

Male *gpt* delta C57BL/6 J mice were administered with various doses of TiO_2_ NPs weekly for 4 consecutive weeks. The long-term mutagenic effects on the liver were analyzed using *gpt* and Spi^−^ mutation assays 90 days after the final injection. We also quantified the amount of titanium in the liver using inductively coupled plasma mass spectrometry and observed the localization of TiO_2_ NPs in the liver using transmission electron microscopy. Although TiO_2_ NPs were found in the liver cells, the *gpt* and Spi^−^ mutation frequencies in the liver were not significantly increased by the TiO_2_ NP administration.

**Conclusions:**

These results clearly show that TiO_2_ NPs have no mutagenic effects on the liver, even though the particles remain in the liver long-term.

## Introduction

Titanium dioxide nanoparticles (TiO_2_ NPs) have become widely used in several industrial applications. Ultrafine TiO_2_ NPs (10–50 nm) cause lung cancer in rats through chronic inhalation [1]. Therefore, TiO_2_ NPs are classified as an IARC Group 2B carcinogen (possibly carcinogenic to humans) [1, 2]. Genotoxicity is one of the key factors to assess the carcinogenic risk to humans. Several studies in mice and rats have reported conflicting results of various genotoxic endpoint analyses [3–16]. Recently, we reported that TiO_2_ NPs have no genotoxic effects in the liver and erythrocytes when intravenously injected into *gpt* delta mice [17], but there are still reports about their positive effect [18, 19]. Thus, the genotoxicity of TiO_2_ NPs remains unclear [20, 21].

TiO_2_ NPs in the rodent bloodstream are translocated to the liver and remain in the tissue long-term [22–25]. However, the long-term genotoxic effects of TiO_2_ NPs in the liver are not well understood. To investigate the long-term genotoxic effects, the mutagenicity is the optimum endpoint among various genotoxic endpoints because of the accumulation potential of TiO_2_ NPs. In this study, we examined the long-term mutagenicity of TiO_2_ NPs and the amount and localization of the remaining particles in the liver after intravenous injection in *gpt* delta mice [26]. Our results indicated that TiO_2_ NPs show no mutagenicity in the tissue, although they remain within the liver cells for an extended period of time.

## Materials and methods

### Animals and reagents

The guidelines for the care and use of laboratory animals set forth by the Institutional Animal Care and Use Committee of the Japan National Institute of Occupational Safety and Health were followed. Male C57BL/6 J *gpt* delta mice were obtained from Japan SLC (Shizuoka, Japan). They were housed under specific pathogen-free conditions with a 12 h light-dark cycle and provided tap water and sterile CE-2 pellets (CLEA Japan Inc., Tokyo, Japan) ad libitum. Aeroxide® P25 titanium dioxide (TiO_2_-P25) was purchased from Sigma-Aldrich (St. Louis, MO, USA).

### Preparation of the TiO_2_ NP suspension and its administration to mice

The TiO_2_-P25 suspension was prepared as previously described [17]. Eight-week-old male *gpt* delta mice were randomly divided into four groups, with 6 mice per group. Mice were administrated by tail vein injection with the TiO_2_-P25 NP suspension at doses of 2, 10, and 50 mg/kg body weight once a week for 4 consecutive weeks. Mice were euthanized on day 90 after the final injection of TiO_2_-P25. Portions of the middle liver lobe were removed for *gpt* and Spi^−^ mutation assays, quantification of titanium by inductively coupled plasma mass spectrometry (ICP-MS), and observation of TiO_2_-P25 particles by transmission electron microscopy (TEM).

### *gpt* and Spi^−^ mutation assay

The *gpt* and Spi^−^ mutation assays were conducted as previously described [17].

### Quantification of titanium in the liver by ICP-MS

Liver samples were weighed and digested with nitric acid and hydrogen peroxide. The concentration of titanium in each sample was measured using ICP-MS (Agilent7900 ICP-MS, Agilent Technologies, Tokyo, Japan). The titanium concentration was determined at the mass number of 47 m/z as previously reported [27].

### Observation of hepatocyte ultrastructure by TEM

Liver samples taken at day 90 after the final injection of TiO_2_-P25 were analyzed using a JEM-2100F transmission electron microscope (JEOL, Tokyo, Japan) as previously described [17].

### Statistical analysis

Statistical significance was examined using Dunnett’s test after one-way ANOVA. Values of *P* < 0.05 were considered significant.

## Results

### Characterization of TiO_2_ suspensions

TiO_2_-P25 was dispersed in disodium phosphate by sonication, as previously reported [17], then diluted to the concentration corresponding to each dose. The hydrodynamic sizes of TiO_2_-P25 in these diluents were measured each time before the injection by dynamic light scattering, and the average of four time determinations was showed in Table 1. The Z-average of the TiO_2_-P25 particles in suspension was about 150 d.nm, regardless of the different concentrations in these diluents.

**Table 1:** Agglomeration sizes of different concentrations of TiO_2_-P25 suspensions in 2 mg/mL disodium phosphate

Concentration (mg/mL)	Dose (mg/kg b.w.)	Z-average (d.nm^a^) mean ± SD	PdI^b^ mean ± SD
0.4	2	153.6 ± 6.5	0.168 ± 0.03
2	10	152.6 ± 7.2	0.146 ± 0.01
10	50	148.0 ± 6.0	0.172 ± 0.01

^a^*d.nm* nm of diameter

^b^*PdI* Polydispersity index

### General observations of the animals

The body weight of mice in each group did not differ at weekly measurement for the first 4 weeks and thereafter to the end of the experiment (data not shown). However, one mouse was dead immediately after the injection at the third week, but the cause for the death could not be identified. Some of the mice got astounded for a short time immediately after the injection, but the mice in all groups were basically not found with abnormal behaviors and appearance.

### Mutation frequencies of *gpt* and Spi^−^ in the liver

The *gpt* and Spi^−^ mutation frequencies in the liver were determined on day 90 after the last administration of TiO_2_-P25 (Tables 2 and 3). Either the *gpt* or Spi^−^ mutation frequencies were not significantly different between the vehicle control group and TiO_2_ administration groups at any dose. These results suggest that TiO_2_-P25 has no mutagenic effect on hepatocytes in mice at 90 d after the last administration.

**Table 2:** The *gpt* mutation frequency in the livers of mice administered TiO_2_-P25 90 days after the last administration

TiO_2_-P25	Total population	Number of mutations	Mutation frequency (× 10^−6^)	
				Mean ± SD
0 mg/kg	1,005,000	1	1.00	
	1,215,000	5	4.12	
	996,000	2	2.01	
	1,242,000	4	3.22	
	1,074,000	4	3.72	
	1,530,000	5	3.27	2.89 ± 1.17
2 mg/kg	1,065,000	7	6.57	
	468,000	8	17.09	
	1,230,000	4	3.25	
	1,089,000	1	0.92	
	1,446,000	4	2.77	
	1,167,000	6	5.14	5.96 ± 5.80
10 mg/kg	846,000	3	6.57	
	912,000	5	3.55	
	711,000	2	5.48	
	771,000	4	2.81	
	1,506,000	2	5.19	
	1,380,000	5	1.33	3.66 ± 1.54
50 mg/kg	717,000	6	8.37	
	552,000	5	9.06	
	909,000	3	3.30	
	1,011,000	5	4.95	
	1,314,000	4	3.04	5.74 ± 2.82

**Table 3:** The Spi^−^ mutant frequency in the livers of mice administered TiO_2_-P25 90 d after the last administration

TiO_2_-P25	Total population	Number of mutants	Mutant frequency (×10^−5^)	
				Mean ± SD
0 mg/kg	1,113,000	14	1.26	
	1,326,000	20	1.51	
	513,000	10	1.95	
	1,185,000	13	1.10	
	951,000	24	2.52	
	1,035,000	14	1.35	1.61 ± 0.53
2 mg/kg	594,000	16	2.69	
	972,000	17	1.75	
	855,000	14	1.64	
	774,000	13	1.68	
	1,344,000	33	2.46	
	1,023,000	20	1.96	2.03 ± 0.43
10 mg/kg	663,000	10	1.51	
	738,000	10	1.36	
	1,404,000	16	1.14	
	1,584,000	17	1.07	
	1,440,000	80	5.56	
	738,000	11	1.49	2.02 ± 1.74
50 mg/kg	1,332,000	20	1.50	
	345,000	1	0.29	
	1,572,000	14	0.89	
	1,026,000	17	1.66	
	915,000	17	1.86	1.24 ± 0.64

### Quantification and localization of TiO_2_ NPs in the liver

**Fig. 1:**
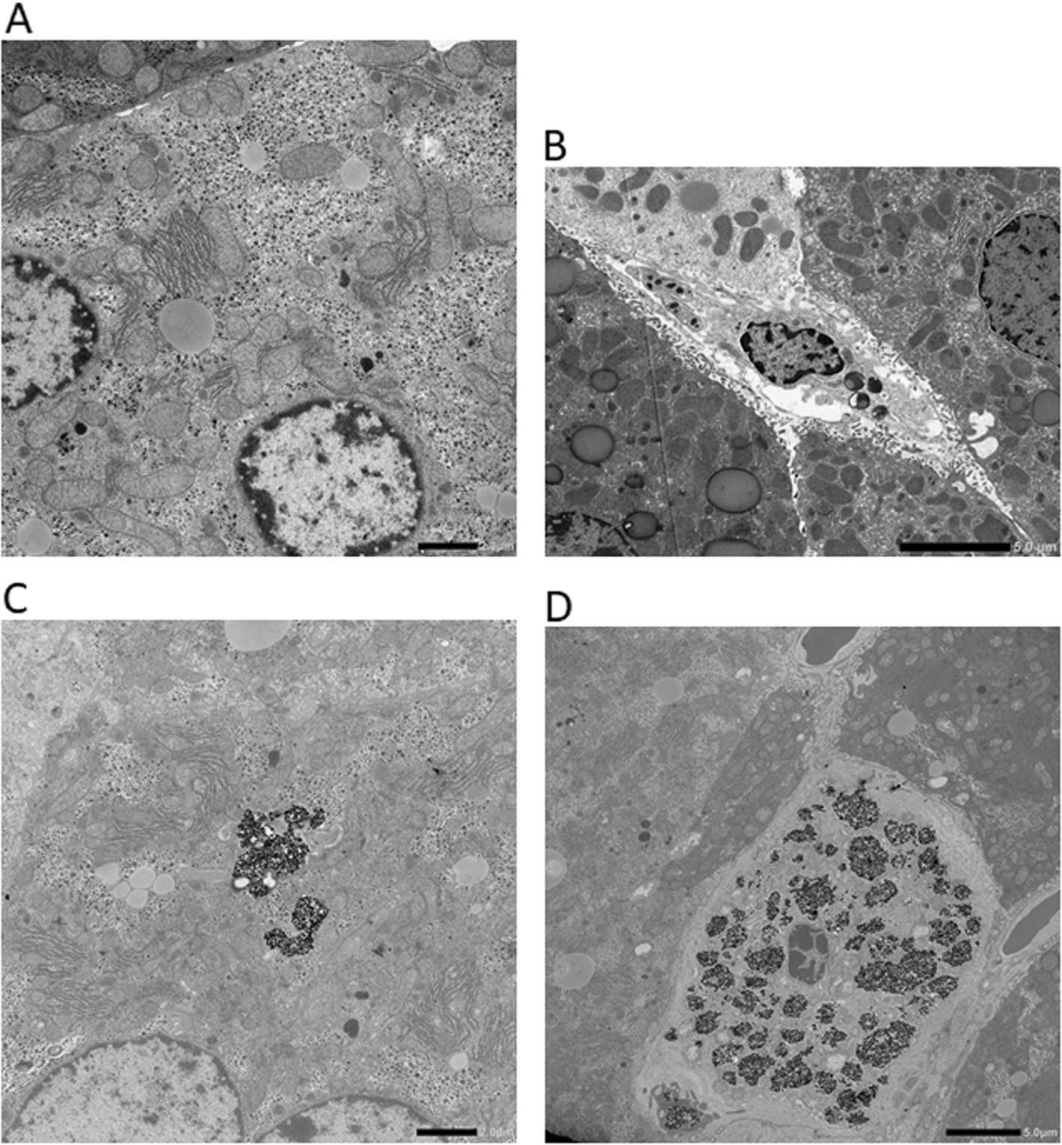
Transmission electron microscope images of mice liver. **a** and **b**, parenchymal hepatocyte and phagocyte, respectively, from the liver of control mice, and **c** and **d**, parenchymal hepatocyte and phagocyte, respectively, from mice administered with 50 mg/kg TiO_2_-P25. * Photo B was from a control mouse in the short period experiment with TiO_2_-P25 (Ref. no.17)

The amount of titanium in the liver of mice administered TiO_2_-P25 was quantified via ICP-MS. The average amount of titanium in the liver was dose dependent (Table 4). To clarify the localization of TiO_2_ particles, liver sections were obtained from mice treated with 50 mg/kg TiO_2_-P25, and the sections were observed by TEM. Large clusters containing the TiO_2_ NPs were found in the parenchymal hepatocytes (Fig. 1c) and Kupffer cells (Fig. 1b), although the clusters were much more prevalent in the latter. The particles were extremely agglomerated within the cytoplasm of both cell types. These results indicate that TiO_2_ NPs remained in the liver 90 d after the last injection and were mainly localized in the cytoplasm of Kupffer cells.

**Table 4:** The amount of titanium in the livers of mice administered TiO_2_-P25 90 days after the last administration

TiO_2_-P25	Analyzed no. of mice	Titanium (μg/g tissue)
0 mg/kg	6	0.10 ± 0.05
2 mg/kg	6	6.9 ± 4.1
10 mg/kg	6	16.0 ± 4.0 *
50 mg/kg	5	24.4 ± 9.1 *

The data are expressed as mean ± SD

Statistical analysis was conducted by Dunnett’s test; *P < 0.01

## Discussion

TiO_2_ NPs have been classified as an IARC Group 2B potential carcinogen. Therefore, their genotoxicity is an important property for risk assessment. Several in vivo studies related to genotoxic effects of TiO_2_ NPs have been reported, but their results are inconsistent [3–17]. Almost all of these reports analyzed the micronuclei and DNA damage by the comet assay, which reveals transient genotoxic consequences that occur shortly after exposure. In addition, the mutagenicity in TiO_2_-accumulating tissues such as the liver and spleen has been examined in transgenic mice for a relatively brief period [8, 17]. Thus, the long-term mutagenic effects of TiO_2_ NPs remain unclear. In this study, long-term genotoxic effects were examined in the liver of mice intravenously administered TiO_2_ NPs.

Recently, we reported that TiO_2_-P25 exhibits no mutagenicity in the liver of mice 9 d after the final injection based on *gpt* and Spi^−^ mutation assays [17]. However, TiO_2_ NPs translocated to the liver are known to accumulate for a long period [22–25]. Thus, we have examined the mutagenicity of TiO_2_-P25 in the liver of long-term housed mice after the last administration. TiO_2_-P25 caused no mutagenic effects in the liver 90 d after the final injection, even though the particles were observed in the liver cells. The state of the cells that incorporated TiO_2_ NPs after 90 d seemed mostly unchanged compared to that after 9 d [17]. In addition, the amount of titanium in the liver at 90 d after the last administration was similar to that at 9 d [17]. This result indicates that TiO_2_ NPs are not easily removed from the liver, even after a long period, but their presence does not cause any adverse effects.

There were a few in vivo studies reported the positive genotoxic effect of TiO_2_-NPs. For example, TiO_2_ (Aeroxide P25®) (same material as used in our present study) induced micronuclei and DNA strand breaks in peripheral blood in adult male mice exposed to 500 mg/kg TiO_2_ NPs of 21 nm size through drinking water for 5 d [14], but the effect was not analyzed in liver. With the same material and intravenous injection route, Dobrzynska et al [4] detected increase of micronuclei in bone marrow polychromatic erythrocytes of mice only at 24 h but not later at 7 and 28 d. Modrzynska et al [28] investigated the DNA strand breaks in the liver of mice treated with TiO_2_ NPs (NanoAmor, 10.5 nm) by intratracheal instillation, intravenous injection or oral gavage at a single dose of 162 μg/mouse, and did not find DNA damages in liver tissue on day 1, 28 or 180 after the exposure by any administration routes, though there was a significant increase in the level of DNA damages in lung tissue on day 180 following intratracheal instillation. These reports suggested that TiO_2_ NPs may induce transient DNA damages in tissue such as blood cells, but not in liver. The endpoint of genotoxicity in this study was gene mutations, which are basically not repairable and could indicate the long term effect. The negative findings in this study are supported by many other reports [6–9, 12, 28]. It is known that physicochemical characteristics (primary size, shape, etc.) and study designs (dose, dispersion method, recovery time, models) can influence the toxicity of nanoparticles in the assay system. More studies with different TiO_2_ NPs are needed to better understand the health effects of this new material.

## Conclusion

TiO_2_ NPs accumulate in the liver cells for long term. However, they do not induce genotoxic effect in the tissue. Therefore, the long-term genotoxic effects of TiO_2_ NPs administered by inhalation and ingestion which may introduce a small portion of the particles into liver, may be negligible in the liver.

## Data Availability

The datasets generated and /or analyzed during the current study are available from the corresponding author on reasonable request.
