# Correction to: Genotoxicity assessment of titanium dioxide nanoparticle accumulation of 90 days in the liver of *gpt* delta transgenic mice

**DOI:** 10.1186/s41021-020-00151-5

**Published:** 2020-03-03

**Authors:** Tetsuya Suzuki, Nobuhiko Miura, Rieko Hojo, Yukie Yanagiba, Megumi Suda, Tatsuya Hasegawa, Muneyuki Miyagawa, Rui-Sheng Wang

**Affiliations:** 1 Division of Industrial Toxicology and Health Effects Research, National Institute of Occupational Safety and Health, 6-21-1 Nagao, Tama-ku, Kawasaki, Kanagawa 214-8585 Japan; 2 Present address: Graduate School of Biomedical and Health Sciences, Hiroshima University, Hiroshima, 734-8553 Japan; 3 Present Address: Department of Health Science, Yokohama University of Pharmacy, Yokohama, 245-0066 Japan; 4 Division of Human Environmental Science, Mount Fuji Research Institute, Yamanashi Prefectural Government, 5597-1 Kenmarubi, Kamiyoshida, Fujiyoshida, Yamanashi, 403-0005 Japan; 5 Present Address: Department of Sport and Medical Science, Faculty of Medical Technology, Teikyo University, Hachioji, Tokyo, 192-0835 Japan

**Correction to: Genes Environ (2020) 42:7**

**https://doi.org/10.1186/s41021-020-0146-3**

In the original publication of this article [1], the author made a correction but wasn’t carried out. The (Fig. 1b) in sentence “*Large clusters containing the TiO NPs were found in the parenchymal hepatocytes (Fig. 1c) and Kupffer cells (Fig. 1b), although the clusters were much more*” should be (Fig. 1d).

The original publication has been corrected.
